# Readability and quality assessment of internet-based patient education materials related to nasal septoplasty

**DOI:** 10.1186/s40463-021-00507-z

**Published:** 2021-03-17

**Authors:** Elysia M. Grose, Connor P. Holmes, Kaishan A. Aravinthan, Vincent Wu, John M. Lee

**Affiliations:** 1 Faculty of Medicine, University of Ottawa, Ottawa, ON Canada; 2 College of Medicine, University of Saskatchewan, Saskatoon, SK Canada; 3 Division of Rhinology, Department of Otolaryngology – Head & Neck Surgery, St. Michael’s Hospital, University of Toronto, Toronto, ON Canada

**Keywords:** Septoplasty, Nasal septum deviation, Quality assessment, Readability assessment, Patient education, Internet-based education

## Abstract

**Background:**

Given that nasal septoplasty is a common procedure in otolaryngology – head and neck surgery, the objective of this study was to evaluate the quality and readability of online patient education materials on septoplasty.

**Methods:**

A Google search was performed using eight different search terms related to septoplasty. Six different tools were used to assess the readability of included patient education materials. These included the Flesch-Kincaid Grade Level, Flesch Reading Ease, Gunning-Fog Index, Simple Measure of Gobbledygook Index, Coleman-Liau Index, and Automated Readability Index. The DISCERN tool was used to assess quality and reliability.

**Results:**

Eighty-five online patient education materials were included. The average Flesch-Reading Ease score for all patient education materials was 54.9 ± 11.5, indicating they were fairly difficult to read. The average reading grade level was 10.5 ± 2.0, which is higher than the recommended reading level for patient education materials. The mean DISCERN score was 42.9 ± 10.5 and 42% (36/85) of articles had DISCERN scores less than 39, corresponding to poor or very poor quality.

**Conclusion:**

The majority of online patient education materials on septoplasty are written above the recommended reading levels and have significant deficiencies in terms of their quality and reliability. Clinicians and patients should be aware of the shortcomings of these resources and consider the impact they may have on patients’ decision making.

**Supplementary Information:**

The online version contains supplementary material available at 10.1186/s40463-021-00507-z.

## Background

Nasal septoplasty is considered the definitive treatment for patients with septal deviation and is one of the most common procedures performed by otolaryngologists – head and neck surgeons [1–3]. Septoplasty not only corrects septal deviation but also improves access and visualization during endoscopic sinus surgery [4]. Despite being a common procedure, patient satisfaction rates after septoplasty vary from 65 to 80%, suggesting not all patients are completely satisfied with the results [5]. Furthermore, the procedure carries a risk of complications including septal perforation, septal hematoma, and synechiae [6, 7]. Thus, patient education is critical to ensuring patients understand the benefits, risks, and expected outcomes after septoplasty.

Patients commonly use the internet to supplement information provided by their physician, with one in five patients using the internet to obtain medical information prior to their medical appointment [8, 9]. However, it is estimated that nearly half of Canadian and American adults have limited ability to understand and act upon health information [10, 11]. Thus, even if online materials are evidence-based and accurate, they can be limited by the use of medical jargon and other technical language, which make them difficult for patients with limited medical knowledge to comprehend. It has been estimated that the average American adult reads at an eighth grade level, and thus the American Medical Association (AMA) currently recommends that patient education materials (PEMs) be written for a grade six level audience [12]. However, studies have shown that online PEMs are often written at a much higher literacy level [13–16]. Within the realm of otolaryngology-head and neck surgery (OHNS), several studies have revealed that online information on OHNS procedures and conditions are written above the recommended grade level and are lacking in terms of quality [17–23]. Since septoplasty is a common OHNS procedure, it is important that clinicians evaluate the information patients are accessing online about their surgery. Thus, the objective of this study was to assess the quality and readability of the PEMs available online related to nasal septoplasty.

## Methodology

### Search

This study did not require Research Ethics Board approval, given the publicly available nature of the information. The search was conducted using Google (www.google.ca) in Ottawa, Ontario on May 1st, 2020. Google was chosen as it is the most commonly used search engine in North America [24]. Prior to initiating the search, all search history, cache, and cookies were cleared. Furthermore, the location settings were disabled and the search was performed using Google Chrome in incognito mode. This was done to minimize the influence of previous search history and location on the search results [25]. Eight search terms were used: “septoplasty”, “septoplasty patient information”, “deviated septum surgery”, “deviated septum surgery patient information”, “nasal septum surgery”, “nasal septum surgery patient information”, “nasal septum repair”, “nasal septum repair patient information”. The first 50 search results from each search term were reviewed.

### Inclusion and exclusion criteria

All search results that were PEMs about septoplasty were included. Exclusion criteria included: websites not written in English, websites where the content was not accessible, audiovisual material, blogs, scientific webpages and articles (i.e. PubMed), webpages geared toward medical professionals, advertisements, websites containing less than 100 words of patient information, and websites without patient information pertaining to septoplasty.

### Categorization of sources

The results were categorized into six categories based on whether they originated from: 1) academic institutions, 2) private medical clinics, 3) professional organizations, 4) government websites, 5) medical information websites (i.e. WebMD) and 6) other miscellaneous sources (i.e. Wikipedia).

### Outcome measures

#### Readability evaluation

Microsoft Word (Microsoft Corp, Redmond, WA, USA) was used to edit the text from included webpages. All formatting elements were removed in this editing process. An online calculator (https://readable.com/) was used to evaluate readability. The following scores were used to assess readability: Flesch Reading Ease (FRE), Flesch Kincaid Grade Level (FKG), Gunning-Fog Index (GFI), Coleman-Liau Index (CLI), Simple Measure of Gobbledygook Index (SMOG), and Automated Readability Index (ARI) [26–28]. Table 1 shows the formula used to calculate the scores [29]. This study employed a comprehensive selection of readability formulas that allowed us to account for several different parameters which impact readability such as word count, syllables, letters per 100 words and sentences per 100 words. Similarly, many previous studies have included these same readability indices when assessing PEMs [20, 21, 23, 26, 27, 30, 31].

**Table 1:** Instruments and calculations used to assess readability

Assessment Scale	Formula
FRE	FRE = 206.835 − (84.6 × $\frac{syllables}{words}$) − (1.015 × $\frac{words}{sentences}$)
FKG	FKG = (11.8 × $\frac{syllables}{words}$) + (0.39 × $\frac{words}{sentences}$) − 15.59
GFI	GFI = 0.4 × [($\frac{words}{sentences}$) + ($\frac{\sigma }{words}$ × 100)] *σ*=number of words with greater than or equal to 3 syllables
CLI	CLI = (0.0588 × *γ*) − (0.296 × *ω*) − 15.8 *γ*=average number of letters per 100 words *ω*=average number of sentences per 100 words
SMOG	SMOG = 1.0430 × $\sqrt{\sigma \times \frac{30}{number\ of\ sentences}}$ + 3.1291 *σ*=number of words greater than or equal to 3 syllables
ARI	ARI = (4.17× $\frac{characters}{words}$) + (0.5 × $\frac{words}{sentences}$) − 21.43

*FRE* Flesch Reading Ease, *FKG* Flesch Kincaid Grade Level, *GFI* Gunning-Fog Index, *CLI* Coleman-Liau Index, *SMOG* Simple Measure of Gobbledygook Index, *ARI* Automated Readability Index

The FKG, GFI, CLI, SMOG, and ARI measured the academic grade level necessary to comprehend the text, with a higher grade level corresponding to text that was more difficult to read. For example, an FKG score of 6 suggests that one would need to have a sixth-grade reading level in order to comprehend the text. The FRE scores ranged from a 0 to 100 with a higher score corresponding to text that is easier to read (Table 2) [32].

**Table 2:** Flesch Reading Ease Score Interpretation

Score	Interpretation
0- < 30	Very difficult
30- < 50	Difficult
50- < 60	Fairly difficulty
60- < 70	Standard
70- < 80	Fairly easy
80- < 90	Easy
90–100	Very easy

#### Quality patient education material

DISCERN is a tool used to evaluate the quality of PEMs [33]. The DISCERN tool consists of 16 questions, which each assess specific criteria. All questions are graded on a scale of 1 through 5 with a score of 1 indicating that the criteria was not met, a score of 2 to 4 indicating that the criteria was partially met, and a score of 5 indicating the criteria was fully fulfilled [33]. The DISCERN tool is further divided into two distinct sections. Reliability is assessed with questions 1 to 8 in the DISCERN tool. The quality of the information on treatment options is assessed with questions 9 to 15. Question 16 is an overall rating of the publication. Total DISCERN scores were calculated from the sum of scores on the 16 questions with a possible range from 15 to 80. Table 3 describes the interpretation of the total DISCERN scores [34]. Two raters (C.H, K.A) independently evaluated the DISCERN scores for each included PEM. Discrepancies were resolved by a third reviewer (E.G). The average scores of the final reconciled DISCERN scores are reported.

**Table 3:** DISCERN Scores

Score Range	Quality Rating
> 62	Excellent
51- < 62	Good
39- < 51	Fair
27- < 39	Poor
< 27	Very Poor

### Statistical analyses

Frequencies and proportions were used to report categorical variables, whereas means and standard deviations were used for continuous variables. Separate analyses were conducted to determine if quality and readability differed depending on the origin of the PEMs. These were compared using the Kruskal Wallis test, followed by Dunn-Bonferroni post hoc tests. The weighted kappa (κ) statistic was used to determine interrater reliability for the DISCERN scoring. Statistical analyses were performed using SPSS (v26.0, IBM Corp, Armonk, NY, USA), with statistical significance set to *p* < 0.05.

## Results

### Search results

Four hundred web pages were retrieved from the search. After the removal of 249 duplicates and excluding 66 web pages, 85 PEMs met the inclusion criteria. Sixty six percent (56/85) of the PEMs originated from the United States, 9.4% (8/85) were from Canada, 11% (9/85) were from the UK, and 14% (12/85) were from other countries. Of the included PEMs, 42% (36/85) originated from academic institutions, 32% (27/85) from private medical clinics, 2.3% (2/85) from professional organizations, 14% (12/85) from medical information websites, 3.5% (3/85) from government websites, and 5.9% (5/85) from miscellaneous sources. Forty-five percent (38/85) of web pages appeared in the search results for multiple search terms.

### Readability

**Fig. 1:**
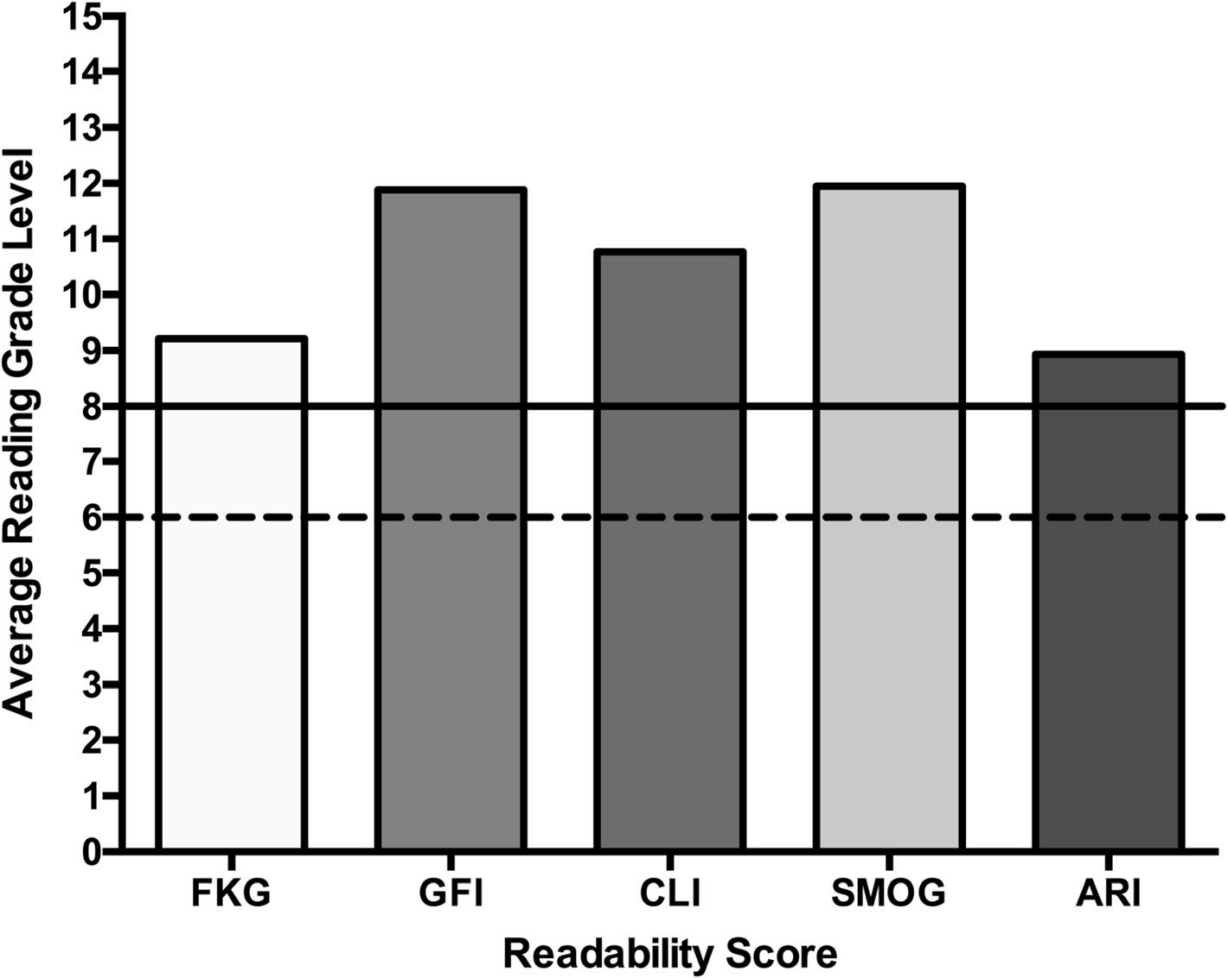
Average Reading Grade Levels. The solid black line represents the eighth grade reading level and the dashed black line represents the sixth grade reading level. FKG: Flesch-Kincaid Grade Level, GFI: Gunning-Fog Index, CLI: Coleman-Liau Index, SMOG: Simple Measure of Gobbledygook Index, ARI: Automated Readability Index

The mean FRE score for all included PEMs was 54.9 ± 11.5 with a range of 35.1 to 78.3. Sixty-eight percent (58/85) had FRE scores below 60. The mean reading grade levels as determined by the FKG, GFI, CLI, SMOG, and ARI scores are displayed in Fig. 1. The average reading grade level determined by all five scores was 10.5 ± 2.0. Table 4 demonstrates the mean readability scores from each source across all six readability indices stratified by the origin of the PEM. PEMs from miscellaneous sources had the highest reading grade levels across all six unique readability indices. PEMs from government websites had the lowest average reading grade levels (Table 4). PEMs originating from academic institutions had significantly higher FRE scores (*p* = 0.002) and lower reading grade levels than PEMs originating from private clinics according to the FKG (p = 0.002), GFI (*p* = 0.003), CLI (p = 0.002), SMOG (*p* = 0.009), and ARI (*p* = 0.005). PEMs from academic institutions had significantly lower reading grade levels than those originating from miscellaneous sources according to the SMOG (*p* = 0.04) and GFI (*p* = 0.03).

**Table 4:** Mean readability scores with standard deviations according to origin

	Academic Institutions	Private Clinics	Professional Organizations	Medical Information Websites	Government Websites	Miscellaneous
Mean FRE Score (SD)	60.1 (11.4)	49.1 (7.6)	50.2 (2.8)	55.4 (13.1)	61.9 (14.3)	46.6 (9.0)
Mean FKG Score (SD)	8.2 (2.0)	10.3 (1.7)	9.5 (0.4)	9.1 (2.3)	7.6 (2.1)	10.9 (1.1)
Mean GFI Score (SD)	10.9 (2.1)	13.0 (1.8)	12.2 (1.0)	11.8 (2.3)	10.2 (2.2)	13.9 (0.8)
Mean CLI (SD)	9.9 (2.1)	11.7 (1.3)	11.6 (0.3)	10.9 (2.1)	9.5 (2.5)	12.1 (1.3)
Mean SMOG (SD)	11.3 (1.6)	12.7 (1.3)	12.4 (0.1)	11.8 (1.7)	10.8 (1.9)	13.4 (0.6)
Mean ARI (SD)	7.9 (2.4)	10.1 (2.1)	8.9 (0)	9.0 (2.5)	6.9 (2.2)	10.8 (0.9)

*FRE* Flesch Reading Ease, *FKG* Flesch-Kincaid Grade Level, *GFI* Gunning-Fog Index, *CLI* Coleman-Liau Index, *SMOG* Simple Measure of Gobbledygook Index, *ARI* Automated Readability Index

### Discern

**Fig. 2:**
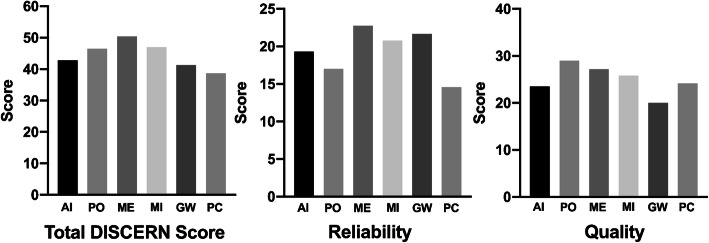
DISCERN rating of PEMs categorized by origin. The overall average DISCERN score, reliability score, and quality scores are shown. AI: Academic Institutions, PO: Professional Organizations, ME: Medical Information Websites, MI: Miscellaneous, GW: Government Websites, PC: Private Clinics

The mean total DISCERN score was 42.9 ± 10.5. The weighted κ statistic for total DISCERN scores was 0.95. Each question included in the DISCERN instrument is scored from 1 to 5 and the average score for each question is displayed in Table 5. Forty-two percent (36/85) of articles had total DISCERN scores less than 39, indicating they were of “poor” or “very poor” quality. Figure 2 demonstrates the DISCERN scores for the PEMs based on their origin. PEMs originating from academic institutions had significantly higher reliability scores than those originating from private clinics (*p* = 0.017). Additionally, PEMs originating from medical information websites had significantly higher reliability scores than those from private clinics (p = 0.002).

**Table 5:** Average score for each item in the DISCERN instrument

Quality Criterion	Mean Rating^a^ (SD)
**Section 1: Reliability**	
1. Are the aims clear?	2.0 (1.4)
2. Does it achieve its aims?	4.2 (0.8)
3. Is it relevant?	3.4 (0.8)
4. Is it clear what sources of information were used to compile the publication (other than the author or producer)?	1.5 (1.1)
5. Is it clear when the information used or reported in the publication was produced?	1.8 (1.1)
6. Is it balanced and unbiased?	3.1 (0.9)
7. Does it provide details of additional sources of support and information?	2.2 (1.3)
8. Does it refer to areas of uncertainty?	2.6 (1.2)
**Total Reliability Score**	18.4 (5.8)
**Section 2: Quality**	
9. Does it describe how each treatment works?	4.5 (1.2)
10.Does it describe the benefits of each treatment?	4.1 (1.4)
11. Does it describe the risks of each treatment?	3.2 (1.9)
12. Does it describe what would happen if no treatment is used?	2.3 (1.5)
13. Does it describe how the treatment choices affect overall quality of life?	2.7 (1.1)
14. Is it clear that there may be more than one possible treatment choice?	2.5 (1.7)
15. Does it provide support for shared decision-making?	2.5 (1.1)
**Total Quality Score**	24.4 (6.8)
16. Overall Rating of Sites	2.7 (1.1)
**Total DISCERN Scores**	42.9 (10.5)

^a^Each question in the DISCERN instrument is scored from 1 to 5. The mean rating represents the mean score for each question in the DISCERN instrument for all the included PEMs

## Discussion

It has been shown that providing information leaflets prior to septoplasty resulted in a positive impact on patient understanding of their procedure when compared to verbal instructions [35]. Being knowledgeable about their treatment plans results in patients taking a more active role in decision making and has been shown to improve outcomes [36, 37]. Thus, we hypothesize that written information regarding septoplasty is critical in facilitating the shared decision-making process and increasing patient satisfaction. However, much of the written information patients are accessing about their treatments originates from the internet. Although this information is widely accessible, it remains largely unregulated resulting in health information of variable quality and credibility as demonstrated by several other authors [20, 21, 38, 39]. Furthermore, even websites that provide high quality, evidence-based information, need to be written at a level appropriate for patients without extensive medical knowledge. In addition to quality, the readability of PEMs is an important consideration to ensure patients can understand and apply information related to their conditions and treatments.

PEMs on septoplasty were above the recommended grade level across all six readability indices used. Given that each readability index accounts for different criteria, the use of multiple indices strengthens this finding. On average, PEMs on septoplasty were written at approximately a tenth grade reading level, which exceeds both the sixth grade reading level recommendation from the American Medical Association and the eighth grade reading level of the average American adult [12]. Furthermore, 68% had FRE scores below 60 indicating that they were “fairly difficult” to “very difficult” to read. Similarly, Cherla et al. conducted a study on the readability of online PEMs on endoscopic sinus surgery and found that over 95% of the material assessed was written above the sixth grade reading level [39]. Even PEMs from the American Rhinologic Society were written between ninth grade and graduate reading level [26]. This is an important finding as some may assume that material originating from credible sources such as those from academic institutions and professional organizations may be better for patient education. This study found that PEMs originating from academic institutions were significantly easier to read than those originating from private clinics and miscellaneous sources. It has been suggested that these differences in readability may be due to the fact that academic institutions may benefit from their affiliations with libraries and other multidisciplinary professionals which in turn improves the delivery of health information [39]. Although academic institutions may produce more patient-friendly materials, our study still found they were above the sixth grade recommended reading level for PEMs. Some suggestions for improving the readability of PEMs include minimizing the use of complex words, decreasing the number of words per sentence and syllables per word, using numbering or bullet points, and writing in an active voice [20, 26, 27]. The authors have created an example patient brochure which adheres to the readability standards discussed in this paper with approximately a sixth grade reading level (Supplementary Material 1).

In addition to readability, PEMs must also contain reliable, comprehensive, and evidence-based information in order to be useful for patient education. In order to assess these aspects, this study evaluated all PEMs with the DISCERN tool. Forty-two percent (36/85) of PEMs had total DISCERN scores corresponding to “poor” or “very poor” quality. Seymour et al. demonstrated similar results when evaluating the quality of web-based patient information on cochlear implantation which revealed that 63% of websites scored as “poor” or “very poor” quality based on total DISCERN scores [19]. In addition to overall scores, looking at the subdomains and individual questions within the DISCERN tool, can highlight more specific deficiencies in the PEMs. Interestingly, the mean total reliability score (based on Questions 1–8) was 18.4 out of a maximum score of 40 whereas the mean total quality score (based on Questions 9–15) was 24.4 out of a maximum score of 35. The differences are likely attributable to questions 1, 4, and 5 with mean scores of 2.0, 1.5, and 1.8, respectively. Question 1 assesses whether the PEM had clear aims. This study found the majority of PEMs on septoplasty did not define who and what they were intended for. A good quality publication has clear aims that help the reader judge whether or not a resource is likely to contain information they are looking for and for who it would be most useful. Furthermore, the majority of PEMs included in this study did not report the evidence used to compile the information (question 4) nor did they include an indication of how current the information was (question 5). Similar findings were demonstrated by Bojrab et al. when they evaluated online information on Ménière’s disease and found DISCERN scores of 1.85 and 2.18 for questions 4 and 5, respectively [38]. Ensuring that authors of PEMs provide clear bibliographies and include revision dates would improve the reliability of these resources. Interestingly, this study also found academic institutions and medical information websites had significantly higher reliability scores when compared to private clinics. These differences may be due to the fact that academic institutions and medical information websites have access to a number of experts in their respective fields and may have more resources to produce more robust PEMs. These findings may have important implications when physicians refer patients to online resources to learn more about septoplasty [40]. The brochure created by the authors (Supplementary Material 1) provides an example of a PEM that was designed to adhere to the quality standards outlined by the DISCERN instrument and score 4 or higher on each component of the DISCERN instrument.

This study has several limitations. Firstly, the search strategy in this study used the Google search engine with eight different search terms to appropriately simulate how patients search the internet for health information. It is possible patients could obtain different resources through using other search engines (i.e Yahoo), however, Google is the most common search engine used and has been the sole search engine used in a multitude of other readability analyses [24, 27, 38, 41]. Furthermore, it is not possible to predict which search terms patients will use, however, this study utilized eight different search terms, which were thought to cover the most likely terms to be used by patients. This study focused on information available online and written in English. Many other potentially useful sources of patient information such as videos, information written in other languages, or patient information booklets provided to patients in the clinic were not evaluated. The correlation between readability scores and true reader comprehension cannot be considered to be perfect as readability scores have several limitations. Since these scores are based on variables such as number of syllables or characters per word, they can be skewed by medical terminology like “turbinectomy” or “reconstruction”. They also do not take into account shorter words that are difficult to understand like “septum”. Additionally, a gold standard readability test does not exist for readability assessment of PEMs, however, this study employed multiple readability tests used in previous literature to provide a comprehensive assessment of readability and minimize the overall impact of factors that can skew the scores. Lastly, although the DISCERN tool has been validated and widely applied to patient information on treatment options, it does not directly evaluate the accuracy of the information contained within these PEMs. This is certainly an area that deserves further study, as it has been demonstrated that online information on septoplasty contained on average 42% of the information patients should know prior to undergoing surgery [42].

## Conclusion

This study assessed online PEMs on septoplasty and demonstrated that the majority of PEMs are written at a level above the recommended reading level. Furthermore, this study revealed some deficiencies in both the quality and reliability of internet-based PEMs on septoplasty. The shortcomings of online PEMs should be emphasized to both patients and providers to ensure adequate and appropriate patient education.

## Supplementary Information

**Additional file 1.**

## Data Availability

The datasets used and/or analyzed during the current study are available from the corresponding author on reasonable request.

## Abbreviations

AMA: American Medical Association
PEMs: Patient education materials
OHNS: Otolaryngology-head & neck surgery
FRE: Flesch Reading Ease
FKG: Flesch Kincaid Grade Level
GFI: Gunning-Fog Index
CLI: Coleman-Liau Index
SMOG: Simple Measure of Gobbledygook Index
ARI: Automated Readability Index
