# Supplementary material for: Readability and quality assessment of internet-based patient education materials related to nasal septoplasty
Source: J Otolaryngol Head Neck Surg. 2021 Mar 17;50:16. doi: 10.1186/s40463-021-00507-z (PMC7970817; doi:10.1186/s40463-021-00507-z)
Supplement: Supplementary file 1 — Additional file 1. [file 40463_2021_507_MOESM1_ESM.pdf]

# SEPTOPLASTY: PATIENT INFORMATION

## WHAT IS THIS BROCHURE MEANT TO COVER?

- This brochure is for patients seeking more information about septoplasty.
- This brochure will give readers details on what to expect from surgery.
- It will discuss who surgery would be useful for.
- It is not meant to replace discussions with your doctors.

## WHAT IS A SEPTOPLASTY?

A septoplasty is a surgery to fix a deviated septum. This operation is done inside the nose (1,2).

## WHAT IS THE NASAL SEPTUM AND WHAT DOES IT DO?

The nasal septum divides the right and left sides of the nose. It is made of both cartilage and bone. The job of the septum is to support the nose and direct the flow of air (1,2).

*This brochure was prepared on January 24, 2021 under the guidance of Dr. Vince Wu, Otolaryngology-Head & Neck Surgery resident, and Dr. John M. Lee, the Head of the Division of Rhinology in the Department of Otolaryngology-Head & Neck Surgery at the University of Toronto.*

## WHAT CAUSES A DEVIATED SEPTUM?

The septum may be deviated since birth. It can also become crooked during normal growth or as a result of nasal trauma (1,2).

## COMMON SYMPTOMS THAT MAY BE ASSOCIATED WITH A DEVIATED SEPTUM (1,2)

- Nasal blockage.
- Runny nose.
- Poor sleep.
- Nasal dryness.
- Sinus infections.
- Bleeding.

Surgery can improve symptoms, quality of life, and breathing.

## WHAT ARE THE TREATMENT OPTIONS FOR A DEVIATED NASAL SEPTUM?

- Treatments such as steroid nasal sprays, saline sprays, and antihistamines can be used to improve symptoms of nasal blockage (3).
- Steroid nasal sprays are used to reduce swelling. They are typically well tolerated. They do carry a small risk of irritation of the nose and nose bleeds (4).
- Antihistamines also work to reduce swelling and irritation. They can cause tiredness, dry mouth and dry eyes (1,2).
- Surgery is the only way to fix a crooked septum.
- Surgery is typically offered after a patient has tried other options and if the deviation matches the patient's symptoms.
- If no treatment is used, symptoms may persist.
- It is important to note that many patients have deviated septums with minimal symptoms.
- It is best to speak with your doctor to find a treatment that suits you.

## WHAT ARE THE RISKS OF SURGERY?

While rare, the risks of this surgery include (2):

- Bleeding.
- Infection.
- Hole in the septum.
- Loss of sense of smell.
- Change in the shape of the nose.

It can be difficult to predict the degree of improvement after surgery. Some patients will have other reasons why their nasal blockage continues even after a septoplasty (i.e. allergies).

## WHAT TO EXPECT AFTERWARDS

- Patients usually go home the same day of surgery.
- Your surgeon may or may not use nasal stents after surgery (3).
- Stents are removed by your doctor around 1 week after surgery.
- Some pain is normal after surgery.
- The nose may feel congested for a few weeks after surgery.
- Work can usually be resumed in 1 week.
- Certain pain medications may be given after surgery which prevent you from driving.
- You may wish to discuss with your family and friends about how to go about your activities while you are recovering.

## FOR MORE INFORMATION

Patient information on deviated nasal septums from the American Academy of Otolaryngology-Head and Neck Surgery:https://www.enthealth.org/conditions/deviated-septum/

## REFERENCES

1. Scholes MA, Ramakrishnan VR. Ent Secrets. 4th ed. Philadelphia: Elsevier Health Sciences; 2015
2. Pasha R, Golub JS. Otolaryngology-Head and Neck Surgery: Clinical Reference Guide. 5th ed. Plural Publishing; 2017.
3. Han JK, Stringer SP, Rosenfeld RM, Archer SM, Baker DP, Brown SM, et al. Clinical Consensus Statement: Septoplasty with or without Inferior Turbinate Reduction. Otolaryngol Head Neck Surg. 2015 Nov;153(5):708–20.
4. Chong LY, Head K, Hopkins C, Philpott C, Burton MJ, Schilder AGM. Different types of intranasal steroids for chronic rhinosinusitis. Cochrane Database Syst Rev. 2016 Apr 26;4:CD011993.
